# A Review on 3D Scanners Studies for Producing Customized Orthoses

**DOI:** 10.3390/s24051373

**Published:** 2024-02-20

**Authors:** Rui Silva, Bruna Silva, Cristiana Fernandes, Pedro Morouço, Nuno Alves, António Veloso

**Affiliations:** 1 CIPER, Faculdade de Motricidade Humana, Universidade de Lisboa, Cruz Quebrada Dafundo, 1499-002 Lisbon, Portugal; apveloso@fmh.ulisboa.pt; 2 CDRSP, Polytechnic University of Leiria, 2430-028 Marinha Grande, Portugal; bruna.c.silva@ipleiria.pt (B.S.); cristiana.fernandes@chleiria.min-saude.pt (C.F.); nuno.alves@ipleiria.pt (N.A.); 3 ESECS, Polytechnic University of Leiria, 2411 Leiria, Portugal; pedro.morouco@ipleiria.pt; 4 CIDESD, Research Center in Sports Sciences, Health Sciences and Human Development, 6201-001 Covilhã, Portugal

**Keywords:** 3D scanner, orthoses, photogrammetry, structured light

## Abstract

When a limb suffers a fracture, rupture, or dislocation, it is traditionally immobilized with plaster. This may induce discomfort in the patient, as well as excessive itching and sweating, which creates the growth of bacteria, leading to an unhygienic environment and difficulty in keeping the injury clean during treatment. Furthermore, if the plaster remains for a long period, it may cause lesions in the joints and ligaments. To overcome all of these disadvantages, orthoses have emerged as important medical devices to help patients in rehabilitation, as well as for self-care of deficiencies in clinics and daily life. Traditionally, these devices are produced manually, which is a time-consuming and error-prone method. From another point of view, it is possible to use imageology (X-ray or computed tomography) to scan the human body; a process that may help orthoses manufacturing but which induces radiation to the patient. To overcome this great disadvantage, several types of 3D scanners, without any kind of radiation, have emerged. This article describes the use of various types of scanners capable of digitizing the human body to produce custom orthoses. Studies have shown that photogrammetry is the most used and most suitable 3D scanner for the acquisition of the human body in 3D. With this evolution of technology, it is possible to decrease the scanning time and it will be possible to introduce this technology into clinical environment.

## 1. Introduction

Orthoses are external medical devices designed to support users’ biomechanical needs, significantly contributing to their quality of life [1,2]. They serve as pivotal elements in controlling and restoring the functionality of an injured body part [3,4]. Customized orthoses, tailored to individual measurements, exhibit innovative attributes concerning device ventilation, thereby minimizing heat injuries, pressure wounds, and skin breakage [5,6].

Traditionally, a custom-made orthosis is manufactured using a plaster cast. This conventional practice has several downsides including high plaster consumption, time-intensive processes, being invasive for patients due to the contact of the plaster and prosthetist-orthotist with the patient’s limb, and a lack of data storage for future reference. To circumvent these medical challenges, reverse engineering techniques have been employed, necessitating three-dimensional (3D) geometric data acquisition. An important requirement for orthoses is comfort, which is attained through a high level of customization facilitated by an accurate capture of the patient’s anatomy [7,8]. Given that each patient possesses a unique body geometry, custom-made orthoses have emerged as the “gold standard”, since the geometry of the orthosis is individually adapted for each patient [9,10]. The journey towards acquiring a custom-made orthosis entails several stages including scanning (digitization), importing the scanned data into a computer to create a computer-aided design (CAD) file, modelling, topological optimization, and 3D printing [11,12,13,14]. The digitization phase is a critical component in this process. To generate a reliable CAD file of the limb, the patient is required to remain still for a certain duration during the acquisition process; hence, fast scanning systems are highly desirable [15]. Also, fast scanning systems offer advantages in terms of non-invasiveness, ease of use, and low cost, making them appealing for reconstructing, measuring, and tracking the evolution of human anatomy for clinical applications [16].

In recent years, several types of 3D scanners have been introduced to expedite the manufacture of customized orthoses. Most digitization systems leverage laser scanners (e.g., HandyScan, Faro), structured light (e.g., Vorum, Artec, Sense 3D), photogrammetry software with conventional cameras (e.g., PhotoModeler, 3DSOM, My3DScanner, PhotoScan, 123D Catch, Hypr3D, RhinoPhoto), or a mixture of diverse technologies. These technologies compute a cloud of three-dimensional points of the object, employing the principle of optical triangulation to shape the natural geometry [3,9,12,17,18]. The selection of the most suitable 3D scanner is contingent on the application and the requisite accuracy [19,20].

This review aims to explore the use of 3D scanners on human limbs for creating CAD models for use in orthosis construction. This study further investigates recent advancements in 3D scanning technology and examines how these developments are improving the custom fabrication of orthoses. This includes a focus on increasing the accuracy in capturing the patient’s anatomy, which contributes to enhanced patient outcomes.

## 2. Materials and Methods

To identify the articles that could be included in this review, the searches were carried out between August and September 2023 in the Web of Science and SCOPUS databases. Searches related to 3D scanners (3d scanner, photogrammetry, reverse engineering, optical scan, laser scanning, structure light) combined with terms for orthoses (orthosis, orthoses) and medical devices were performed. No restrictions were applied to the year or type of publication.

Original articles written in English with 3D scanners used to acquire human limbs to make custom orthotics were included. All narrative and systematic reviews and dissertations were excluded. Any articles not written in English were excluded. Any article with another scanner device (ex. computational tomography (CT) or X-ray) were excluded. Articles using 3D scanners other than for obtaining scans of the human body and other than for orthoses were excluded. Articles whose purpose used 3D scanners for prostheses were also excluded.

After the removal of excluded articles and deletion of duplicates based on PRISMA, data extraction was standardized. Titles and abstracts from the search results were screened using the eligibility criteria and reviewed by two authors (R.S. and B.S.) for inclusion. Data extraction and evaluation of the remaining articles were performed independently by the same authors. In case of disagreement, an additional reviewer (P.M.) was consulted. Data extraction included first author and year, and the aim of the study (reverse engineering, 3D scanner and different types, orthoses), among others.

## 3. Results

**Figure 1:**
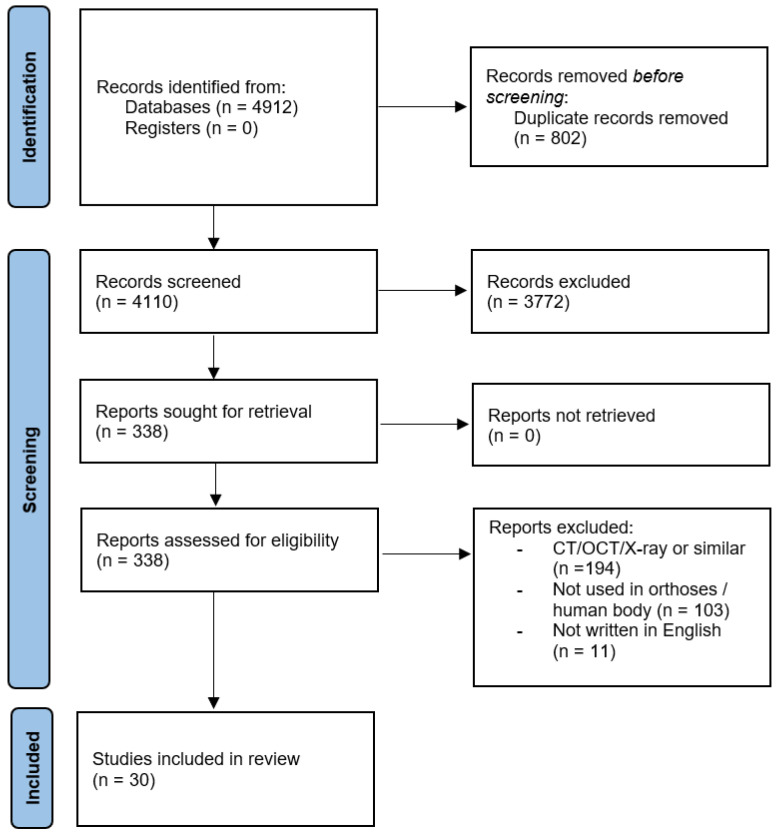
Flow diagram of the search history and selection process.

Figure 1 summarizes the results of the different steps to identify appropriate articles for the review, based on PRISMA guidelines [21]. The initial database search identified 4912 articles, and after duplicate removal, 4110 were considered potentially relevant and were screened for relevant content. No additional articles were identified following a hand-search of the reference lists. After reading the title and abstract of the 4110 articles, 338 were selected for possible inclusion in this systematic review and the full-text articles were retrieved. In the last phase, articles that used CT/OCT/X-ray; that did not use humans in their methodology (ex. Moulds); and that were not written in English were excluded. A total of 30 of the 338 articles were included in this review, organized by the type of 3D scanner that was studied (Table 1).

## 4. Discussion

The production of customized orthotics has increasingly garnered attention, with projections indicating a significant surge in their utilization over the next decade. This growth is primarily attributed to advancements in 3D-scanning technologies, which are becoming faster, simpler, and more effective [35]. The clinical and research applications of 3D scanning systems, particularly in anthropometric measurement, have been well-documented. Considering growing concerns regarding the use of radiation in medical imaging, these systems offer a safer alternative by minimizing patient exposure to radiation, as seen with X-ray or computer tomography.

Despite their potential, the integration of new 3D technologies in the National Health Service remains limited. The main hurdles are the high costs involved, as well as the time and training required for prosthetist-orthotist professionals to adapt to using 3D scanners. Overcoming these challenges could revolutionize the process of supplying customized orthoses, making it more efficient and cost-effective [1]. Several studies have validated the feasibility of Additive Manufacturing (AM) and scanners in producing customized daily living aids. This approach is expected to significantly enhance the quality of life of patients at a reduced cost [1,3,15,19,26,27,29,35,39]. Patients have expressed a preference for scanning over traditional plaster casting methods [1]. The 3D-printed orthoses are noted for their accurate geometrical correspondence to patient anatomy and comfortable fit, striking a balance between precision and affordability [11,24].

### 4.1. Photogrammetric Scanners

Photogrammetry, as a 3D scanning technology, utilizes photographs to create detailed three-dimensional models. This method involves taking multiple photographs of an object from various angles and merging these images to construct a comprehensive 3D representation. Such an approach is particularly valuable in orthotic design, where the accurate replication of body parts is essential. Additionally, photogrammetry eliminates the issue of body movement during scanning and does not require markers on the patient [43]. 

Dal Maso and Cosmi [11] and Ciobanu et al. [22] have effectively demonstrated the utility of photogrammetry in creating detailed and accurate 3D models for orthotic applications. Their research, which focused on ankle-foot orthoses, illustrated the method’s capability in generating high-fidelity scans. These scans were instrumental in producing orthoses that are both well-fitting and comfortable for the wearer. The study particularly highlighted photogrammetry’s strength in capturing intricate details, a critical factor for areas needing precise support. Ciobanu et al. [22] expanded the use of photogrammetry to the creation of foot orthoses. Their findings emphasized photogrammetry’s potential in generating detailed mesh structures, which are crucial for designing orthoses that accurately match a patient’s anatomical structure. They also identified challenges in scanning areas with deep depressions or occlusions, which can affect the precision of the final 3D model. A notable advantage of photogrammetry is its ability to quickly acquire data. This is a significant benefit over other scanning methods that might necessitate extended and static patient positioning. With photogrammetry, a complete scan can be obtained in a relatively short period, thereby reducing patient discomfort and minimizing errors caused by movement [22]. The evolution of photogrammetry software has also played a key role in simplifying the transformation of raw images into usable 3D models. Improvements in image processing algorithms have enabled more accurate model reconstruction, even in suboptimal photographic conditions. This advancement is particularly important in clinical environments where time efficiency and resource optimization are crucial. However, photogrammetry does have its limitations. The need for the precise alignment of images in photogrammetry and the possibility of inaccuracies in regions with complex geometry or poor contrast are challenges that need to be addressed [46]. Moreover, converting STL models to CAD for orthotic design requires a certain level of expertise in both photogrammetry and CAD software [2].

### 4.2. Structured Light Scanners

Structured light scanning, a critical technology in the field of orthotic design, is represented significantly in this review, comprising 13 out of the 30 studies analysed. This prevalence underscores its extensive utilization and importance in the development of orthotic devices. This technology works by projecting patterned light lines from a fixed source (like a projector), capturing detailed coordinates of the scanned model, including colours and textures [47,48,49], and providing high-resolution data crucial for creating detailed orthotic devices.

All the studies concluded that this technology is capable of accurately replicating complex body geometries including fine details, surface textures, and minor anatomical variations, an important aspect in the creation of effective and comfortable orthoses. Furthermore, the authors addressed various challenges associated with structured light scanning. One of the primary limitations noted is the requirement for the subject to remain still during the scanning process. As structured light involves capturing multiple images from different angles in a constant flow, even slight movements can lead to inaccuracies in the final model [50]. This aspect can be particularly challenging when working with certain patient groups, such as children or individuals with certain disabilities. Another consideration is the processing time and computational requirements [51]. While this technology can capture highly detailed data, processing these data into a usable 3D model can be time-consuming and resource intensive. Advances in computing power and software optimization are gradually overcoming these limitations, making structured light scanning more accessible and efficient.

This technology has been tested and analysed on practically every part of the body, ranging from the head and neck [25,30,33], to upper limbs including the hand, [24,25,28,32,34] to lower limbs [15,19,26,27,29,31].

Baronio et al. [24] exemplify the potential of structured light scanning in orthotic fabrication. Their research focused on the creation of spinal orthoses. They highlighted the technology’s ability to capture the complex curvature and nuances of the spine with remarkable precision, a critical factor in designing effective spinal orthoses. There was a particular focus in the cases of Krajňáková et al. [25], Powers et al. [29], and Rogati et al. [19] on determining whether the obtained anatomical model was accurate both metrically and qualitatively. Krajňáková et al.’s [25] study, especially, addresses the limitations in capturing hair and facial hair (beard), a common problem across all technologies. While it is possible to eliminate this interference in certain parts of the human body (for example, using a sock on a lower limb or gloves on upper limbs), in other body parts it may become complicated without some form of prior hair removal. The studies by Rogati et al. [19], Powers et al. [29], and Ambu [30] et al. were the only ones that directly conducted a monetary value comparison between scanners, but they never directly compared them with the traditional plaster casting method.

### 4.3. Laser Scanners

Laser technology is typically employed for scanning shapes and surfaces. It efficiently gathers anthropometric data, aiding in the production of customized orthoses based on digital scans [35,52]. Nonetheless, its limited range can be a disadvantage, particularly for larger body parts like legs and feet, as the process becomes time-consuming [53]. 

Roberts et al.’s [1] study becomes relevant for its comparison between 3D scanners and the Traditional Method Plaster Caster. They tested a considerable sample of lower leg scans to construct 134 AFOs and conducted a double-blind randomized controlled trial. This trial demonstrated that the time for constructing an orthosis using 3D scanners is on average 28.2 min less, and that 70% of patients expressed a preference for being scanned rather than having their limbs cast in plaster. Nonetheless, a significantly higher proportion of scan-based AFOs failed to meet the specifications stipulated by the orthotist, resulting in an increased production time of 9 days. The most recent study employing laser scanning technology dates to 2020, which may indicate a decline in the use of this type of technology for acquiring human models for subsequent orthosis construction via AM.

### 4.4. Optical Scanners

Optical scanners, which project light over the body and trace surface topography, collect data to form a “point cloud”. These data are then processed through computer algorithms to generate a precise model [54]. While these scanners are accurate, they require a balance between the scanning speed and the resolution of their optical and electronic components to produce a clean CAD model [55]. 

Optical scanners tend to be more cost-effective and user-friendly compared to other types. However, they are more susceptible to errors during capture, as they do not emit their own light and are extremely dependent on the quality of the ambient lighting where the acquisition is taking place. Notably, Buonamici et al.’s [40] study constructed a new type of scanner, achieving reconstruction errors in the range of [−2.9, 1.5] mm using Active Stereoscopic technology.

### 4.5. Comparing Technologies

When comparing different technologies, photogrammetry enables rapid capture, but its processing time can be lengthy [43]. For example, Weigert et al. [44] found that, while capturing 62 photos through photogrammetry took only 30 min, the reconstruction process was time-consuming. Belokar, Banga, and Kumar [3] combined laser and structured light technologies, completing a scan in just one minute by manually rotating the scanner around the patient’s limb. Despite the processing times, these methods are still faster than traditional plaster casting. From a global perspective, photogrammetry stands out as one of the most promising options due to its accuracy, minimal acquisition time, high-fidelity colour [43], and shape reproduction [39], although only three studies used this technology. This is probably due to the expensive cost associated with photogrammetry equipment which can often be beyond the means of departments and healthcare professionals. The equipment also lacks accurate calibration. Despite this, low-cost photogrammetry has been increasingly recognized as a feasible and effective method. Particularly Structure-from-Motion photogrammetry has been highlighted as a low-cost and accurate technique for acquiring 3D models of human limbs [56]. The use of low-cost 3D limb-scanning technology has been evaluated for its repeatability and validity in obtaining accurate representations of limb geometry at a reasonable cost [57]. Additionally, smartphone photogrammetry has been investigated, demonstrating the optimization of methods and quantitative evaluation of suitability for prosthetics and orthotics [58]. The integration of photogrammetry with smartphone technology has been explored to facilitate low-cost limb scanning, expanding the scope of orthotic telemedicine and providing affordable limb scans to underserved areas [59]. Moreover, the combination of photogrammetry and transfer learning with DeepLabv3 for image segmentation has been proposed to facilitate low-cost limb scanning using cell phones, further emphasizing the potential for cost-effective applications in orthotics [59]. 

### 4.6. Patient Centred Outcomes

From a biomechanical perspective, the study by Mo et al. [31] compared standard and 3D-printed orthoses created using 3D scanners and AM technologies. They found lower peak rearfoot eversion angles during running with both types of orthoses compared to running barefoot, although no statistical differences were observed between the orthoses. Similarly, Telfer et al. [37,38], employing the same patient data acquisition methodology, demonstrated that customized foot orthoses could provide a dose–response effect for selected plantar pressure variables. However, they found no corresponding effect on muscle activity. They further noted a dose–response effect, with a linear trend for both the rearfoot and knee, in treating the pronated foot type with customized foot orthoses.

In comparisons of AM ankle-foot orthoses (AFO) with conventional ones, patients preferred the AM AFO for its lighter weight and ease of use, despite the conventional AFO being more effective in certain aspects [27]. Notably, four studies [27,32,34,42] employed the Quebec User Evaluation of Satisfaction with Assistive Technology (QUEST) test to evaluate dimensions, weight, adjustments, safety, durability, simplicity, comfort, and effectiveness. Two of these studies involved orthoses designed using 3D structured light scanners, and their findings demonstrate that patients are more satisfied with 3D-printed orthoses than with conventional orthoses. These observations underscore the critical role of patient-centred design in orthotic development, where customization and material choice are pivotal in enhancing the user experience. The integration of evaluation tools like QUEST into clinical practice provides invaluable insights for healthcare professionals, facilitating a deeper understanding of patient needs and preferences. This comprehension is crucial in guiding the selection and design of more effective and comfortable orthotic solutions, particularly in rehabilitation contexts. Such patient-centric approaches in orthotic design not only cater to functional needs but also significantly improve the overall satisfaction and quality of life for the users.

Zheng et al. [41] reported that AM orthoses resulted in better outcomes compared to low-temperature thermoplastic plate orthoses in reducing spasticity and swelling, and in improving motor function and the passive range of wrist extension in stroke patients. Additionally, Lee et al. [34] designed and manufactured a patient-specific assistive device using 3D printing techniques, optimized for the functional needs of a patient with brain injury, after assessing the patient’s disability status. The subsequent step involves transferring the acquired data to CAD software for mesh adjustment and measurement processing. Various reverse engineering softwares like Rhinoceros, Rapidform, Geomagic, and LeiosMesh are used, although this stage is time-consuming and demands high expertise from the user. The challenge lies not only in the orthotist-prosthetist’s proficiency with 3D scanners but also in the user friendliness of these software systems, which are not yet optimally aligned for direct orthosis construction [24,36].

### 4.7. Future Research

Despite the importance of the outcomes provided by most studies on the use of 3D scanners, a notable gap is observed in the detailed description of methodologies, limiting the potential for replication and comparison by other researchers. Many studies lack comprehensive details about scanner characteristics and the types of software used, particularly for orthosis construction. Additionally, one study even fails to identify the scanner or its technology. Moreover, the involvement of actual patients in these studies is limited, with much of the research being conducted on healthy individuals. Considering the anticipated future reliance on these 3D technologies, it becomes imperative to conduct more research within clinical settings.

## 5. Conclusions

Currently, it is possible to capture the human anatomy using 3D scanners. However, reducing the digitization time remains a crucial challenge in order to prevent any minimal movement from the patient. While the results are promising, they also highlight the challenges associated with integrating new technologies into clinical practice. Considerations such as the initial costs of equipment, training requirements, and the need to adapt clinical workflows are significant. Generally, the studies analysed suggest that photogrammetry and structured light are the most suitable 3D-scanning technologies for acquiring human body data for custom orthotics. There is also a growing belief in the field that scientists will increasingly develop 360° 3D scanners capable of capturing the human limb’s anatomy in a single shot. The traditional method of building custom-made orthoses with plaster casts has remained largely unchanged despite the introduction of new technologies which aid post-processing. With today’s advancements, there is an opportunity to transition from the traditional method to one that better meets the needs of patients and professionals. As 3D scanners become more affordable, their integration into clinics becomes feasible, allowing for the proper training of health professionals. However, there is a need to develop specific software to streamline the orthosis building process, making it both easier and faster.

## Figures and Tables

**Table 1:** Included studies with scanners details, anatomical zone, type of software, outcomes, and conclusions.

Reference	Anatomical Zone	3D Scanner			Software Used to Process the Data Acquired		Outcomes	Conclusions
		Name	Type of 3D Scanner	Characteristics of 3D Scanner	Scanner	CAD		
PHOTOGRAMMETRY								
Dal Maso and Cosmi, 2019 [11]	Ankle-Foot	Default Camera	Photogrammetry	No data	Agisoft Photoscan Pro	SolidWorks	150 photos taken; 80% of calculation time was used for photo alignment, tie point cloud, sparse cloud cleaning, and cleaning. Mesh exportation to STL format with ≈20,000 faces.	Scanner can be used on other parts of the body. The printed orthosis had great geometrical correspondence and comfort. The method showed instability when converting STL into CAD which requires experience and ability.
Ciobanu et al., 2013 [22]	Foot	Default Camera	Photogrammetry	No data	3DSOM	No data	From 20 to 40 leg shots were taken. The software automatically created a cloud of 3D points from photos and transformed the points into a 3D mesh.	Use of photogrammetry was feasible in the case of foot orthosis fabrication as a cost-effective 3D reconstruction technique. Some problems in surfaces with indentations and blind holes.
Ranaldo et al., 2022 [23]	Forearm	Structure Sensor Mark II	Photogrammetry	Based on active stereo vision	Autodesk MeshMixer	Rhinoceros 7.0	The analysis was carried out on three cast meshes having different pattern distributions but an identical overall shape. All models show a perfectly elastic behaviour, with a maximum σv well below the tensile strength of the material (50 MPa) and a maximum displacement.	This study shows that a semi-automatic, programmable tool allows designing anatomically customized orthopaedic casts with optimized features for the treatment of forearm fractures. Its main advantages are: it does not require specific CAD skills to perform the design of the orthosis; it does not take significant time for the generation of the model; the designs can be subject to finite element analysis to foresee different load scenarios and validate the choice of geometry.
STRUCTURED LIGHT								
Baronio et al., 2016 [24]	Hand (including fingers)	3D Scan-in-a-Box	Structured light	Scans in about 4 s, with metric accuracy of 0.1% in relation to the size of the object size	No data	Rhinoceros	Anatomy was obtained with 8 acquisitions (2 min each). Total scanning time was 1 h 30 min for acquisition with data cleaning and rigid alignment with 1 h for mesh creation, regularization, and repair.	The methodology was geometrically satisfactory with a favourable trade-off between high accuracy (in the reproduction of the patient anatomy) and low cost requirements.
Krajňáková et al., 2019 [25]	Shoulder, neck and face	Artec EVA	Structured light	Resolution: 0.5 mm; accuracy: 0.1 mm; distance accuracy: 0.03% at 1000 mm; texture resolution: 1.3 Mpx;	Artec Studio	No data	The scanner captured all types of hair. Better results with tousled hair and wet hair. Beard is not advisable, as the neck area will be blurred. It is possible to scan complicated body shapes.	Scanner outputs can be used in medicine for the design and manufacture of orthoses and dental implants; simulation before and after plastic surgery, preservation of cultural heritage, and virtual reality.
Dessery and Pallari, 2018 [26]	Knee brace	Artec EVA and iSense	Structured light	Artec Eva: Resolution: 0.5 mm; frame rate: 16 Hz iSense: Resolution: 0.9 (at 0.5 m) ± 30 mm (at 3 m); frame rate: 30 Hz	Artec Studio 9 (Artec Eva); 3DSizeMe (iSense)	Artec Studio 9 (Artec Eva); MSoft (iSense)	Three scans were performed on each participant with Artec (410 s ± 118 s) and iSense (507 s ± 94 s) from the malleolus to the upper thigh art. Processing time was 3588 s ± 423 s for Artec and 460 s ± 169 s for iSense. Mean circumferences were created to compare results.	Manual measurement is the most accurate method to take lower limb measurements, but the inter- and intra-reliability is poor and information about leg shape is limited. 3D scanners can provide lower limb measurements with similar accuracy, but better repeatability ((intraclass correlation coefficient: 0.99–1.0) and 0.15% mean differences).
Rogati et al., 2019 [19]	Foot	Microsoft Kinect Sensor and IQube	Structured light	Microsoft Kinect: Laser emitter an infrared and an RGB camera to obtain a 300,000 point-cloud Accuracy: 2.8 ± 0.6 mm; IQube: No data	Skanect (Kinect); No data (IQube)	Geomagic	Acquisition time for the Kinect was 25 s. The comparison between 3D scans of the plantar surface resulted in error of 2.8 ± 0.6 mm (left feet) and 2.9 ± 0.4 mm (right feet). In the arch region they were 1.4 ± 0.4 mm (left feet) and 1.6 ± 0.5 mm (right feet). Good repeatability of the Kinect scans was observed. The foot dimensions were like the corresponding PodoBox (manual measurement).	The total cost of the prototype created with the Kinect Sensor is about 200–300€, which is at least one order of magnitude lower than that of commercial laser-based foot scanners. While accuracy and repeatability results were largely consistent across subjects, and between left and right foot intra-subject, the sample of feet analysed was small.
Dombroski, Balsdon and Froats., 2014 [15]	Foot	Microsoft Kinect	Structured light	No data	No data	MeshLab	Two AFOs were created. One by AM with Kinect and one created by TPCM. The TPCM provided the most control over movement of the medial longitudinal arch. The arch height index (AHI) was 21.2 mm (shod only), 21.4 mm (AM AFO) orthosis, and 22.0 mm (TPCM).	The 3D printing AFO resulted in a higher AHI than the shod condition; however, the differences between the 3 conditions were minimal. Variability was similar with standard deviations within 0.13 mm. Sample size of only one subject was small. Kinect could be a low-cost method of custom foot orthotic manufacturing.
Cha et al., (2017) [27]	Ankle-Foot	Artec Eva	Structured light	Maximum snap rate: Up to 16 fps	No data	MediACE 3D	The study compared an AM AFO with conventional AFO. In QUEST, all items were ranked as “very satisfied” or “satisfied.” The patient was more satisfied with the AM AFO regarding weight and ease of use, and more effectiveness was shown on conventional AFO.	The AM AFO focused on the weight, individualization, and comfort rather than the function. In addition, the printed AFO had the advantage of being easily wearable inside a shoe compared to the conventional AFO, which usually requires larger shoes to wear.
Kim et al., 2018 [28]	Wrist and hand	Artec Eva	Structured light	No data	Artec™ Eva	Geomagic Touch and Geomagic Freeform software	The Patient-Rated Wrist Evaluation showed significant pain relief in both groups. Two items of the 28 Orthotics and Prosthetics Users’ Survey (OPUS) questions, “Put toothpaste on brush and brush teeth” and “Dial a touch tone phone”, showed high satisfaction scores, with statistically significant difference in the experimental group.	The 3D-printed wrist orthosis was superior to the cock-up orthosis in some items of the OPUS. Wrist pain was reduced in the 3D-printed wrist orthosis as well as the cock-up orthosis, so the 3D-printed wrist orthosis could possibly play the same role as the off-the shelf cock-up orthosis.
Powers, et al., 2022 [29]	Ankle-Foot	Structure Sensor	Structured light	No data	Design Studio software (Standard Cyborg, Inc., San Francisco CA, USA)	No data	Excellent interrater reliability was obtained for scan-based measures. Excellent intrarater test-retest reliability was established for the scanning process. MDC values for intrarater test-retest reliability were typically around or below 4 mm for foot and ankle measures, and under 6 mm for circumference and length	The results of this study demonstrate that low-cost 3D limb scanning can be used to obtain valid and reliable measurements of 3D limb geometry for the purpose of AFO fitting, when collected using the clinically relevant standardized conditions presented here.
Ambu et al., 2023 [30]	Neck	Microsoft Kinect	Structured light	1 Mpx depth sensor, a 12 Mpx RGB camera and two IR illuminators to obtain mappings of the object’s depth with high accuracy in a very short time. The illuminator used in wide field of-view mode is tilted an additional 1.3 degrees downward relative to the depth camera	No data	No data	Topology Optimization (TO) model, structurally evaluated by means of FE analysis, also in comparison with an orthosis having a ventilation pattern configured as Voronoi cells, showed a satisfactory behaviour; also considered that voids are large for extension and flexion loading, stress distribution occurs in areas of limited size with reference to the extent of the upper parts where the load is applied. The highest values of maximum displacement and maximum Von Mises stress were obtained for extension loading; however, maximum displacement was lower than 2 mm, while maximum stress was under the limit value for HPB.	A scanning system made up of three synchronized low-cost sensors, suitably arranged, has been developed. This system allows a fast acquisition, about 5 s, with minimum discomfort for the patient. The scanning system is also potentially suitable to hospital setting, being low cost and provided with a GUI for semi-automatic management of the device. The manufacturing of prototypes was done with a new bio-based material, which also contributes to lightness and satisfies the aesthetic demands. Neck temperature measurements highlighted a better performance for the TO orthosis even with the insertion of a padding. TO orthosis is very promising as regards user’s comfort, an automatized strategy for the procedure will be investigated.
Mo et al., 2019 [31]	Foot	David SLS HD 3D scanner	Structured light	No data	No data	Geomagic Freeform	Results showed lower peak rearfoot eversion angles during running with TPM or 3D printed (3DP) orthoses than no-orthoses control condition (CON). No differences were observed in other biomechanical parameters among the three conditions. Running with TPM and 3DP orthoses resulted in better perceived comfort in “medial-lateral control” and “heel cushioning” than CON. There were no statistical differences in all parameters between TPM and 3DP orthoses.	The present findings indicate improved comfort during running with TPM or 3DP orthoses, which hinted 3DP orthoses could be a viable alternative to TPM orthoses for clinical practice.
Chu et al., 2020 [32]	Hand	Structure Sensor	Structured light	No data	No data	Mesh Mixer	The QUEST revealed the highest score in the mean satisfaction level. The items evaluated were dimension, weight, adjustments, safety, durability, simplicity, comfort, and effectiveness.	The new process saves time and is highly accurate in clinical practice. The short thumb orthosis prototype created by the proposed design procedure offers satisfactory functional quality in numerous aspects and high practicality in clinical practice.
Kuo et al., 2019 [33]	Head and neck	Go! SCAN 50	Structured light	Max Resolution: 0.5 mm	No data	No data	Smartphone use increased the head and neck flexion angles in all postures, and sitting without back support showed the greatest head and neck flexion angles. The posture-correcting effect of the customized collar was better than the Aspen Vista and Sport-aid collars. In addition, the customized collar was more comfortable to wear than the other two collars in most contact areas.	Smartphone use increased both the head and neck flexion in different postures, and the proposed customized 3D-printed cervical collar significantly reduced the head and neck angles.
Lee et al., 2019 [34]	Hand	Artec Eva	Structured light	No data	No data	Geomagic Freeform	The JHFT score improved after application of 3D-printed devices. In most QUEST items, 3D-printed devices showed better results than ready-made assistive devices. The typing speed became faster in 3D-printed devices than in ready-made assistive devices. The patient was satisfied with the orthosis in writing with a pen, eating food, and typing on a keyboard because of its fitness to his hand and ease of use.	The study designed and manufactured a patient-specific assistive device optimized for patient function after estimating the disability status of a patient with brain injury through 3D-printing techniques.
LASER								
Roberts et al., 2016 [1]	Ankle-Foot	FastSCAN	Laser	No data	No data	Rodin 4D	A total of 134 AFOs fabricated with CAD technology and traditional plaster method in a double-blind randomised (1:1) controlled trial design were compared. No difference in time taken to cast or scan the limbs. Rectification and moulding time for cast AFOs was 55.1 ± 26.0 min and for scanned AFOs was 26.9 ± 12.2 min.	70% of patients said they preferred to be scanned over having their limbs cast in plaster. A significantly higher proportion of scan-based AFOs failed to meet the specification stipulated by the orthotist, increasing production time by 9 days.
Parry, Best and Banks, 2020 [35]	Grip for the Hand	ROMER Absolute Arm	Laser	No data	Geomagic Wrap	Fusion 360 with Nettfab	The data collection was approximately 10 min. Manufacturing time was 10 h 5 min with a cost of €10.90 (with overheads and machine depreciation excluding labour).	The study demonstrated that AM and Scanners is a viable method of producing customised daily living aids, which is anticipated to improve quality of life for sufferers of arthritis at low-cost.
Liu et al., 2019 [36]	Ankle-foot	EinScan—Pro 3D,	Laser	No data	No data	Geomagic Studio	With respect to the temporal-spatial parameters, the velocity and stride length in the gait with AFO increased significantly as compared to the gait without AFO. The cadence increased, double limb support phase decreased, and the step length difference decreased in the gait with AFO; however, the difference was not statistically significant.	This study confirmed the feasibility of patient-specific AFO fabricated by AM techniques and demonstrated the process of modifying AFO models successfully. The specific ankle-foot orthoses fabricated by material PA12 have a significant effect on the improvement of velocity and stride length in people with stroke.
Telfer et al., 2013a [37]	Foot	No data	Laser	No data	No data	No data	Significant group effects were seen with customized FOs reducing above knee muscle activity in pronated foot types compared to normal foot types. Interaction effects were seen for gastrocnemius medialis and soleus. Significant linear effects of posting level were seen for plantar pressure at the lateral rearfoot, midfoot and lateral forefoot. A group effect was also seen for plantar pressure at the medial heel.	This study provides evidence that customized FOs can provide a dose–response effect for selected plantar pressure variables, but no such effect could be identified for muscle activity. Foot type may play an important role in the effect of customized orthoses on activity of muscles above the knee.
Telfer et al., 2013b [38]	Foot	No data	Laser	No data	No data	No data	Significant and linear effects of posting were seen for the peak and mean rearfoot eversions, peak and mean ankle eversion moments, and peak and mean knee adduction moment variables. Group effects were observed for the peak and mean forefoot abduction and for the peak knee adduction moment	These data indicate that a dose–response effect, with a linear trend for both the rearfoot and knee, exists for customized FOs used to treat pronated foot type.
OPTICAL								
Sabyrov et al., 2021 [39]	Neck	Sense (2nd generation)	Optical scanner	No data	No data	Fusion 360	The extended support section, which is positioned on trapezius muscles, improved comfortability, and stability. The breathability of skin is achieved via well-distributed elliptical holes. The convex shape at the front of the model gives convenient swallowing. Application of flexible TPE (flex) material adds flexible property, hence enhancing the dressing process. Comparative to PLA material, it has a lower density, which defines low weight. The negligible deformation during numerical assessment emphasized the strength of design.	The fabricated orthosis model possesses high accuracy in terms of the neck shape of the patient. This was accomplished through 3D scanning and further processing of the CAD model. The advantage and applicability of new cervical orthosis design and the flexible filament were demonstrated.
Buonamici et al., [40]	Arm	Oplà 2.0	Optical scanner	Depth technology: Active stereoscopic Operating range: ~0.16–10 m Resolution: 1280 × 720 Framerate: Up to 90 fps Field of view FOV: H69°, V43°, D77° (±3°)	Oplà 2.0 GUI	No data	All errors measured in the reconstruction were in the range of [−2.9, 1.5] mm, the mean error of the signed distance was −0.49 mm with a standard deviation of 0.64 mm. The composition of the panel group has allowed the validation of the acquisition system on significantly different hand–wrist–arm anatomies.	Except some local errors, Oplà 2.0 performed well within the limits imposed by the accuracy requirements.
Zheng et al., 2020 [41]	Wrist-hand	HCP	Optical scanner	No data	No data	Unigraphics NX 8.0 Software	After six weeks: – A significant difference was found between the two groups (experimental group and control group) in the change of Modified Ashworth Scale scores; – There was no statistically significant difference between the two groups in flexion and radial-deviation angles; – There was a significant difference between the two groups in the change of Fugl-Meyer Assessment scores; – No statistically significant difference was found in the change in visual analogue scale scores between the two groups; – A statistically significant difference was found in the change in swelling scores between the two groups; – No statistically significant difference was found in the change in subjective feeling scores between the two groups.	3D-printed orthosis showed greater changes than low-temperature thermoplastic plate orthosis in reducing spasticity and swelling, improving motor function of the wrist and passive range of wrist extension for stroke patients.
Fu et al., 2022 [42]	Ankle-Foot	Sense (2nd generation)	Optical scanner	No data	No data	Rhinoceros	The study acquired data from 10 hemiplegic stroke participants. Gait performance and plantar pressure for AM AFO, standard AFO, and barefoot on 10 m walking. Plantar pressure of hemiplegic leg increased as in AM AFO compared with bare foot. Contact area and peak pressure increased with AM AFO vs. standard AFO and barefoot. QUEST was made to evaluate participant satisfaction. Mixed results for satisfaction obtained without statistical differences.	Dynamic plantar pressure measurement is feasible and useful for evaluation of ankle equinovarus deformity in hemiplegic stroke patients. AM AFO has at least the same ability to increase medial midfoot plantar pressures over affected leg compared with standard AFO. More medial weight bearing and more symmetric contact area over sole with AM AFO, which is more similar to physiological finding in normal subject.
COMBINATION OF DIFFERENT SCANNERS								
Grazioso et al., 2018 [43]	Spinal	INBODY and Polhemus FastSCAN SCORPION	Photogrammetry and Laser	Photogrammetry: Circular structure, 17 pillars (7 cameras each with 5 Mpx). Polhemus FastSCAN SCORPION: No data	No data	No data	Photogrammetry allowed instantaneous capture, but processing time was longer vs. laser scanner. Deviation between scanners was +0.90 mm and −1.11 mm. The laser scan obtained 13,150 faces and the inbody scan obtained 68,750 faces.	The photogrammetric scanner showed good accuracy and high-fidelity colour. Availability in medical centres could help the patients, thanks to the minimally invasive procedure and medical practitioners, in having a system which is simple to use.
Belokar, Banga and Kumar, 2017 [3]	Ankle-Foot	No data	Laser with structured light	No data	No data	No data	The 3D scanner was rotated manually around patient’s limb to create the template model in just one minute. Time-saving approach when digitizing.	3D scanning is suitable to produce custom orthotics.
Weigert et al., 2016 [44]	Foot	Default Camera and Roland MDX-40	Photogrammetry and Mechanical 3D scanner (Touch Probe/Point-to-point scanner)	Photogrammetry: Sony Xperia SP C5303 smartphone Roland MDX-40: Head course: 305 mm × 305 mm × 105 mm; Accuracy variable and up to 0.04 mm	Scanner: No data (Roland MDX-40); Memento (photogrammetry)	CATIA; Geomagic	A total of 62 photos taken for photogrammetry and reconstruction take 30 min. MDX-40 took 26 H to scan the plaster cast. Relative error between plaster model and photogrammetry was 2.85% and 0.72% between plaster model and MDX-40.	Both scans showed similar topography of the foot. Mechanical presented more irregularities; however, this mesh provided more details that the MDX-40 especially between the toes. The 2.85% relative error presented by photogrammetry could be compensated with the application of soft material on the surface.
Volonghi, Baronio and Signoroni. 2018 [7]	Hand	Cronos 3D Dual (static scanner) and Insight3 (real-time scanner)	Structured light and optical scanner	Cronos 3D Dual: 4 s per frame; 2 Mpx. Accuracy: ±30–60 μm. Insight3: Real time; 1280 × 1024 Px Accuracy: ±0.25–0.5 mm.	Optical RevEng 2.4	No data	Scan processing time was 7.5 min for volunteers and 9 min for patients. Error inferior to 0.5 mm between scanners. Cronos 3D with volunteers achieved a complete scan. Insight3 with patients did not have any motion artifacts.	For Cronos 3D, motion artefacts relating to involuntary movements were successfully corrected. The preservation of all fine textural information of the final aligned model was demonstrated. For Insight 3, motion artefacts were reduced or even avoided. Both scanners proved appropriate for hand anatomy acquisition.
NO SCANNER TYPE SPECIFIED								
Murzac et al., 2021 [45]	Spine	No data	No data	No data	Meshmixer	Fusion 360	The scanning and processing of the obtained data can be done following the procedure described in this paper. This ensures a compliant geometry for virtual analysis of the product that will be produced for a certain user. At the same time, the generative design guarantees the choice of a geometry, manufacturing technologies, and a material that leads to the choice of the optimal option from a technological and economic point of view. Also, with the help of the software filters, it is possible to identify the optimal variant for the manufacturer according to the objectives set for each production cycle	The chosen option can vary from one production batch to another and from one stage of the product life cycle to another. The final concept of the spinal orthosis is designed for upper body posture correction of clinically healthy individuals, with no pre-existing congenital malformations of the spine.

TPCM—Traditional Plaster Casting Method; Mpx—Megapixels; Px—Pixels; fps—frames per second; Hz—Hertz; QUEST—Quebec User Evaluation of Assistive Technology; AM—Additive Manufacturing; AFO—Ankle Foot Orthosis; m—meters; h—hours; FO—Foot Orthosis; PA12—Polyamide 12 (Nylon 12); TPM—Traditional Plaster Method; TPE—Thermoplastic Elastomer; TPU—Thermoplastic Polyurethane; PLA—Polylactic Acid; CAD—Computer-Aided Design.
